# Metagenomic Analysis Reveals Variations in Gut Microbiomes of the *Schistosoma mansoni*-Transmitting Snails *Biomphalaria straminea* and *Biomphalaria glabrata*

**DOI:** 10.3390/microorganisms11102419

**Published:** 2023-09-28

**Authors:** Peipei Li, Jinni Hong, Mingrou Wu, Zhanhong Yuan, Dinghao Li, Zhongdao Wu, Xi Sun, Datao Lin

**Affiliations:** 1 Department of Parasitology, Zhongshan School of Medicine, Sun Yat-Sen University, Guangzhou 510080, Chinawuzhd@mail.sysu.edu.cn (Z.W.); 2 Key Laboratory of Tropical Disease Control, Ministry of Education, Sun Yat-Sen University, Guangzhou 510080, China; 3 Provincial Engineering Technology Research Center for Diseases-Vectors Control, Sun Yat-Sen University, Guangzhou 510080, China; 4 Department of Traditional Chinese Medicine, Guangdong Provincial People’s Hospital, Guangdong Academy of Medical Sciences, Southern Medical University, Guangzhou 510180, China

**Keywords:** *Schistosoma mansoni*, intermediate host, metagenomics, gut microbiota, variation pattern

## Abstract

*Biomphalaria* snails play a crucial role in the transmission of the human blood fluke *Schistosoma mansoni*. The gut microbiota of intermediate hosts is known to influence their physiological functions, but little is known about its composition and role in *Biomphalaria* snails. To gain insights into the biological characteristics of these freshwater intermediate hosts, we conducted metagenomic sequencing on *Biomphalaria straminea* and *B. glabrata* to investigate variations in their gut microbiota. This study revealed that the dominant members of the gut microbiota in *B. glabrata* belong to the phyla Bacteroidetes and Proteobacteria, which were also found to be the top two most abundant gut bacteria in *B. straminea*. We identified Firmicutes, *Acidovorax* and *Bosea* as distinctive gut microbes in *B. straminea*, while *Aeromonas*, *Cloacibacterium* and *Chryseobacterium* were found to be dependent features of the *B. glabrata* gut microbiota. We observed significant differences in the community structures and bacterial functions of the gut microbiota between the two host species. Notably, we found a distinctive richness of antibiotic resistance genes (ARGs) associated with various classes of antibiotics, including bacitracin, chloramphenicol, tetracycline, sulfonamide, penicillin, cephalosporin_ii and cephalosporin_i, fluoroquinolone, aminoglycoside, beta-lactam, multidrug and trimethoprim, in the digestive tracts of the snails. Furthermore, this study revealed the potential correlations between snail gut microbiota and the infection rate of *S. mansoni* using Spearman correlation analysis. Through metagenomic analysis, our study provided new insights into the gut microbiota of *Biomphalaria* snails and how it is influenced by host species, thereby enhancing our understanding of variant patterns of gut microbial communities in intermediate hosts. Our findings may contribute to future studies on gastropod–microbe interactions and may provide valuable knowledge for developing snail control strategies to combat schistosomiasis in the future.

## 1. Background

Schistosomiasis is a significant helminthic disease caused by infection with the blood flukes of the genus *Schistosoma*. It is considered one of the most important diseases in humans, infecting over 230 million people worldwide [1,2]. Endemic in 77 countries in tropical and subtropical regions, schistosomiasis poses a threat to approximately one-eighth of the global population. The disease is caused by different species of *Schistosoma*, including *Schistosoma mansoni* [3,4,5], *S. japonicum* [6,7,8,9], *S. haematobium* [10,11], *S. intercalatum* [12] and *S. Mekongi* [13,14,15], of which *S. mansoni* is one of the most widespread species [2,3]. *Biomphalaria* snails such as *Biomphalaria straminea* and *B. glabrata* are important intermediate hosts of *S. mansoni*. In China, *S. japonicum* is the only endemic parasitic flatworm that causes schistosomiasis. However, with the fast spread of the invasive vector *B. straminea*, there is the potential risk of *S. mansoni* transmission in South China [4,5]. Therefore, it is crucial to focus on managing the spread of this invasive vector and gaining a better understanding of the biological characteristics of intermediate hosts like *B. straminea* and *B. glabrata*.

The gut microbiota plays a crucial role in the health of organisms [16]. It has been shown to influence various aspects of host physiology and contribute to host fitness [16,17,18,19,20,21]. The composition of gut microbiota can be influenced by numerous factors, including host biology, host genetics, diet, ecological niche and developmental stages [22,23,24,25,26,27]. Recent studies using 16S rRNA gene sequencing have shown the diverse gut microbiota to be associated with different gut sections, sexes and developmental stages in intermediate hosts such as *Pomacea canaliculata* and *Achatina fulica*, which are hosts to *Angiostrongylus cantonensis* [28,29,30,31,32,33]. However, there is limited knowledge regarding the gut bacteria of *S. mansoni* intermediate hosts like *B. straminea* and *B. glabrata* and the relationship between the gut microbiota and biology of these hosts.

Metagenomics analysis, in contrast to 16S rRNA gene sequencing, offers alternative methods for identifying uncultivated microbes and obtaining more functional information about specific microbes in organisms such as humans, ruminants and mice [34,35,36,37]. However, few studies have focused on the variations in composition, functions and antibiotic resistance genes (ARGs) in the gut microbiota of *S. mansoni* intermediate hosts.

In this study, we utilized metagenomic sequencing to analyze the gut microbiota of *B. straminea* and *B. glabrata*, and we compared the influence of gut microbiota affected by host species. Our findings contribute to a better understanding of the basic characteristics of gut-microbiota–gastropod associations and may provide new insights for the development of interventions against schistosomiasis.

## 2. Methods

### 2.1. Samples Collection

*B. straminea* snails were reared under laboratory conditions at Sun Yat-sen University. The snails were kept under controlled conditions, with a temperature of 25 to 27 °C, 80% relative humidity (RH) and a 12:12 h light–dark (L:D) cycle. The snails were fed sterile food. The laboratory population of *B. straminea* was collected from Shenzhen, China, and has been reared for over 10 years under laboratory conditions. *B. glabrata* was introduced from the University of Bristol School of Veterinary Sciences, UK, [38] and has been reared for over 10 years under laboratory conditions. The *S. mansoni* Puerto Rican strain was provided by the Institute of Tropical Medicine, Nagasaki University, Japan [38].

Before dissection, the snails were surface-sterilized in sterile water for 30 s and washed three times in different sterile dishes. Then, they were rinsed in sterile phosphate-buffered saline (PBS) for 15 s. To obtain the soft tissue of gastropod snails, the shells were removed from the snails using sterile metal tweezers, and the soft tissues of the snails were cleaned with sterile PBS for 15 s and repeated two times. After the above procedures, snail soft tissue was placed in a sterile dish and dissected using sterile glass rods. All the procedures were performed under sterile conditions. The gut samples were stored at −80 °C for further studies.

### 2.2. DNA Extraction

Each gut sample from the snails consisted of 10–15 guts pooled together. Each collected gut sample was homogenized in a tube using a sterile pestle (an electric tissue homogenizer) on ice. Total DNA was extracted from the gut samples using a HiPure MicroBiome DNA kit (Magen, Guangzhou, China), following the manufacturer’s protocol. The extracted DNA was resuspended in 50 μL AE buffer and stored at −80 °C until further study. The DNA quality and quantity were assessed using a Nanodrop (Thermo Scientific, Waltham, MA, USA). Total DNA degradation and potential contamination were monitored on 1% agarose gels. Total DNA concentration and purity were monitored on a Qubit 2.0 Fluorometer (Invitrogen, Carlsbad, CA, USA) and a NanoPhotometer^®^ spectrophotometer (IMPLEN, Westlake Village, CA, USA).

### 2.3. Metagenomic Sequencing, Quality Control, Assembly and Annotation

Total DNA (1 μg/sample) was used as input material for the DNA preparations. The genomic DNA was arbitrarily fragmented into 350 bp in size. PCR products were purified using the AMPure XP system. The libraries were analyzed for size distribution by an Agilent2100 Bioanalyzer, quantified using real-time PCR and, finally, sequenced on an Illumina NovaSeq 6000 platform (Novogene, Beijing, China). Paired-end reads were generated.

The raw metagenomic sequences were quality-controlled prior to the assembly. Sequencing adapters were filtered using SeqPrep (https://github.com/jstjohn/SeqPrep/) (accessed on 29 September 2022). Low-quality reads (with a quality value <20 or length <50 bp or having N bases) were removed. The remaining reads were aligned to the snail genome using BWA (v. 0.7.9a) [39], which is available at http://bio-bwa.sourceforge.net (accessed on 29 September 2022). After alignment, any hits associated with the reads and their corresponding mate pairs were removed. Clean reads were assembled using SOAPdenovo software (http://soap.genomics.org.cn/) (accessed on 29 September 2022). Scaffolds with a length of over 500 bp were extracted. Finally, contigs with a length ≥300 bp were used for prediction and annotation, and contigs were then subjected to open reading frame (ORF) prediction using MetaGene (http://metagene.cb.k.u-tokyo.ac.jp/) (accessed on 29 September 2022) [40]. The predicted ORFs were retrieved and translated into amino acid sequences using the NCBI database (http://www.ncbi.nlm.nih.gov/Taxonomy/taxonomyhome.html/) (accessed on 29 September 2022).

The representative sequences of the nonredundant gene catalog were aligned to the NR database using Diamond (http://www.diamondsearch.org/index.php, (accessed on 29 September 2022) v. 0.8.35), and an e-value cutoff of 1 × 10^−5^ was applied during the alignment process for taxonomic annotations [41]. KEGG annotation was performed using BLAST against the Kyoto Encyclopedia of Genes and Genomes database (http://www.genome.jp/keeg/) (accessed on 29 September 2022). ARGs in reads were identified based on the ARDB database (http://ardb.cbcb.umd.edu/) (accessed on 29 September 2022).

### 2.4. Bacterial Data Analysis

Principal coordinates analysis (PCoA) based on Bray–Curtis distance as the measure of beta diversity and the similarity among samples was performed in R software (Version 2.15.3) [42]. NMDS analysis based on Jaccard distance was performed using the online tool Majorbio Cloud Platform. Taxonomic microbes were identified by using the linear discriminant analysis (LDA) effect size (LEfSe) [43]. Analysis of similarities (ANOSIM) was used to evaluate the beta diversity using mothur in R. Heatmap generation based on color gradation was carried out in Excel. Statistical analysis of metagenomic profiles (STAMP) and correlation network analysis showing the correlation between gut microbe species were performed on the Tutools platform (https://www.cloudtutu.com/) (accessed on 18 December 2022). The relationships among variables were analyzed using Spearman analysis in R. The credibility of statistical analysis is represented by *p*-value. All pairwise comparisons for each two groups were analyzed by using the Wilcoxon test to range adjustment, with a *p*-value threshold of 0.05.

### 2.5. Parasite Detection

The *Biomphalaria* snails were reared in a laboratory setting following the methods outlined in the previous study [4,17]. Each snail was exposed to 10 *S. mansoni* miracidia; the specific procedures for infecting the snails with miracidia were detailed in the study [5]. The snails that were exposed to *S. mansoni* were subject to shading treatment throughout the experiment. The infection rate was assessed 80 days after cercariae infection using the methodology described in the previous study [44]. The release of cercariae from *Biomphalaria* snails was previously documented in the research [5].

### 2.6. Statistical Analysis

The data are expressed as the mean ± standard error of the mean (SEM). The difference between groups was analyzed by *t*-test. *p* < 0.05 is considered statistically significant.

## 3. Results

### 3.1. The Composition of Gut Microbes of B. straminea and B. glabrata

**Figure 1:**
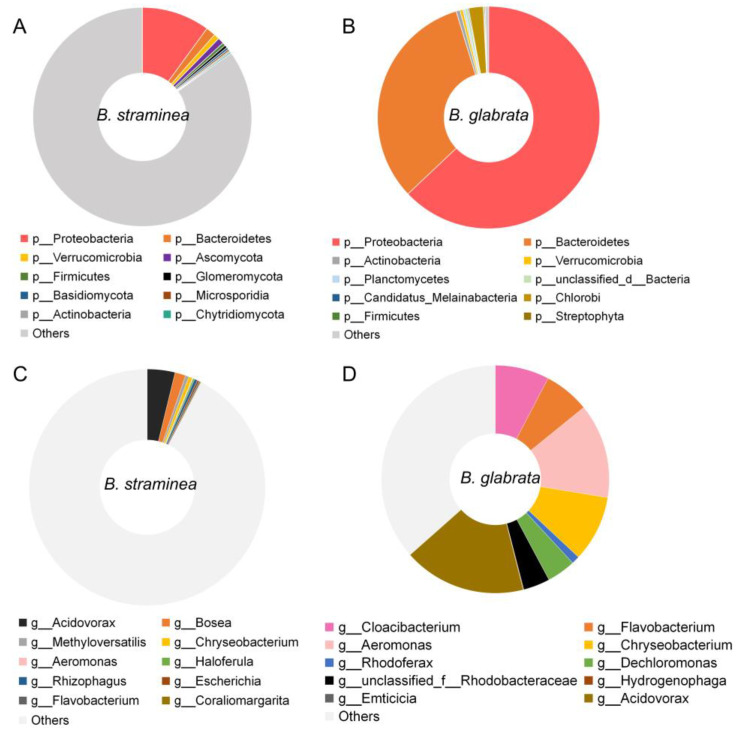
The relative abundance of gut bacteria of *B. straminea* (n = 4) and *B. glabrata* (n = 5): at the phylum level of *B. straminea* (**A**) and *B. glabrata* (**B**) and at the genus level of *B. straminea* (**C**) and *B. glabrata* (**D**). The top ten most common gut microbes are shown.

Metagenomic analysis generated a total of 58.8 Gb of raw data from nine samples, with five samples from *B. glabrata* and four samples from *B. straminea*. The results showed that the majority of the gut microbial community of *B. glabrata* corresponded to members of the phylum Proteobacteria (63.6%), followed by Bacteroidetes (36.7%) (Figure 1A). In *B. straminea*, the relative abundance of Proteobacteria (10.0%) and Bacteroidetes (1.4%) was lower compared to *B. glabrata* (Figure 1A).

At the class level, Betaproteobacteria was found to be the most abundant gut bacteria in both *B. straminea* and *B. glabrata* (Figure S1A). At the order level, Flavobacteriales, Burkholderiales and Rhodocyclales were the dominant gut bacteria in *B. glabrata*, while Burkholderiales was the most abundant gut microbiota of *B. straminea* (Figure S1B). At the family level, *B. glabrata* had three dominant families, Weeksellaceae, Comamonadaceae and Azonexaceae, which were different from the composition of *B. straminea* (Figure S1C).

At the genus level, the gut bacterial composition of *B. straminea* showed 10 genera, with *Acidovorax* and *Bosea* being the dominant ones. In comparison, *B. glabrata* exhibited six dominant gut microbes, including *Dechloromonas*, *Acidovorax*, *Aeromonas*, *Cloacibacterium*, *Flavobacterium* and *Chryseobacterium* (Figure 1B). These findings highlighted that the genus *Acidovorax* is a shared dominant microbe in guts of both *B. straminea* and *B. glabrata.* At the species level, the top five gut microbes of *B. straminea* include *Bosea* sp. AAP35, *Bosea vaviloviae*, *Haloferula* sp. BvORR071, *Rhizophagus irregularis* and *Methyloversatilis discipulorum*, and gut bacteria of *B. glabrata* with an average relative abundance >2% include the species *Cloacibacterium rupense*, *Rhodobacteraceae bacterium* PARR1, *Acidovorax temperans*, unclassified *Aeromonas* and *Aeromonas jandaei* (Figure S1D).

In a previous study, the authors defined the core microbiota of the human gut as taxa that were present in at least 50% of samples, and they considered these taxa to be representative of the microbial community that is commonly found in the human gut [45]. In this study, among the top 10 features (Figure 1), Bacteroidetes, Proteobacteria, *Acidovorax*, *Aeromonas*, *Chryseobacterium* and *Flavobacterium* were present, based on their average relative abundance, throughout the different sample types and defined as “core” microbes of the snail guts. Based on network analysis, the “core” gut microbes of both *B. straminea* and *B. glabrata* were analyzed. At the phylum level, the phylum Bacteroidetes directly associates with multiple bacteria such as Candidatus Kapabacteria, Candidatus Curtissbacteria, Uroviricota and Balneolaeota. Similarly, the phylum Proteobacteria shows a positive association with Gemmatimonadetes, which correlates significantly with 14 microbes including Nitrospinae and Rotifera (Figure S2A). At the genus level, *Acidovorax, Aeromonas* and *Chryseobacterium* are directly associated with various microbes belonging to different clusters in the network (Figure S2B).

### 3.2. Microbiome Composition Shifts Associated with Host Species

**Figure 2:**
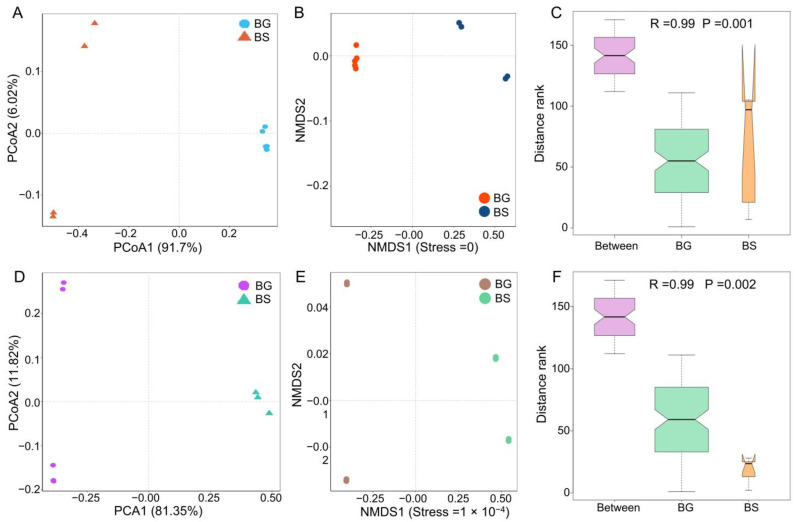
The differences in community structures of gut bacteria between *B. straminea* (n = 4) and *B. glabrata* (n = 5). (**A**) PCoA analysis. (**B**) NMDS. (**C**) ANOSIM. (**D**) PCoA analysis. (**E**) NMDS. (**F**) ANOSIM. Pictures (**A**–**C**) show the difference in bacterial community structure based on the phylum level. Pictures (**D**–**F**) show the difference in bacterial community structure based on the genus level. BG: *B. glabrata*. BS: *B. straminea*.

To investigate the factors shaping the gut microbiota, we compared the microbiome composition between host species. Our analysis revealed distinct segregation of bacterial community structures based on host species at the phylum level, as shown by PCoA analysis using the Bray–Curtis distance (Figure 2A). The gut bacteria formed clusters specific to each host species, indicating that the host species can influence the configuration of the gut microbiota in intermediate hosts, as is evident from the NMDS analysis (Figure 2B). Furthermore, the host species significantly impacted the composition of gut microbes in intermediate hosts, as indicated by the R statistic obtained from the analysis of similarities (ANOSIM: *p* = 0.001) (Figure 2C). Similar results were observed at the genus level (Figure 2D–F).

**Figure 3:**
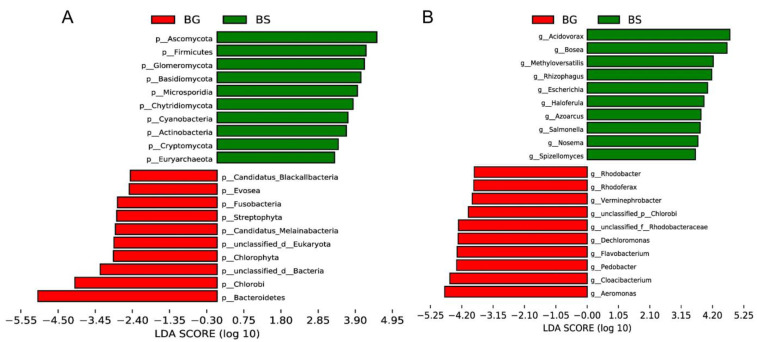
A linear discriminant analysis (LDA) effect size (LEfSe) analysis of gut microbiota of *B. straminea* (n = 4) and *B. glabrata* (n = 5): (**A**) at the phylum level, and (**B**) at the genus level. The top 20 most different gut microbes are shown. BG: *B. glabrata*. BS: *B. straminea*.

We conducted further analysis on different sample populations and identified Firmicutes, *Acidovorax* and *Bosea* as characteristic gut microbiota features in *B. straminea*. On the other hand, gut microbes such as Bacteroidetes, *Aeromonas*, *Cloacibacterium* and *Chryseobacterium* were identified as *B. glabrata*-dependent features (Figure 3). These results suggested that a range of gut bacterial features can serve as biomarkers to distinguish *B. straminea* and *B. glabrata*.

### 3.3. Bacterial Functional Shifts Associated with Host Species

**Figure 4:**
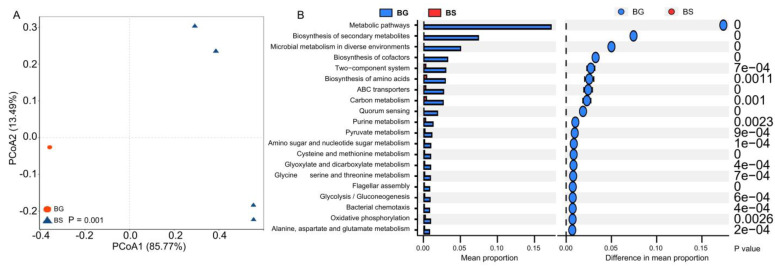
The difference in gut bacterial functions of *B. straminea* (n = 4) and *B. glabrata* (n = 5). (**A**) PCoA analysis. (**B**) STAMP analysis. The top 20 most significantly different functions of *B. glabrata* and *B. straminea* gut microbiota are shown, respectively. BG: *B. glabrata*. BS: *B. straminea*.

Gut microbial metabolism functions play a crucial role in various aspects in the host organisms [46,47,48]. To further investigate the associations between gut microbial functions and shaping factors, we performed a metagenomic analysis. The functional profiles of gut bacteria (at level 3) from different host species were found to cluster separately, indicating that the host species significantly influences the metabolic configuration of the gut microbiota (Figure 4A). Furthermore, based on the statistical analysis of metagenomic profile (STAMP) analysis, we observed a significantly higher abundance of functions related to metabolic pathways, biosynthesis of secondary metabolites, ABC transporters and two-component systems in the gut microbiota of *B. glabrata* compared to *B. straminea* (Figure 4B). These findings suggest that snail species can have a substantial impact on the functional diversity and metabolic capabilities of the gut microbiota.

### 3.4. The Distribution of ARGs of the Gut Microbiota of B. straminea and B. glabrata

Antibiotic resistance genes (ARGs) have become a global public health concern [49,50]. In our study, we investigated the distribution of ARGs of the gut microbiota of *B. straminea* and *B. glabrata*. We identified the presence of ARGs associated with various classes of antibiotics, including bacitracin, chloramphenicol, tetracycline, sulfonamide, penicillin, cephalosporin_ii and cephalosporin_i, fluoroquinolone, aminoglycoside, beta-lactam, multidrug and trimethoprim in snail guts (Figure S3A). The dominant ARGs were those related to bacitracin for both *B. straminea* and *B. glabrata*. Furthermore, the abundance of ARGs associated with various classes of antibiotics, including bacitracin, chloramphenicol, tetracycline, sulfonamide, aminoglycoside, beta-lactam, multidrug and trimethoprim, was higher in *B. glabrata* compared to *B. straminea* (Figure S3A).

Additionally, we identified a total of 46 antibiotic resistance types, such as *baca*, bl1_*asba*, bl1_*fox*, bl3_*cpha*, bl3_l, *ceob* and *cml*_e3, present in either *B. straminea* or *B. glabrata* (Figure S3B). The most dominant antibiotic resistance type observed in both species was *baca*. Furthermore, the majority of antibiotic resistance types found in *B. glabrata* were higher than those in *B. straminea* (Figure S3B). These findings suggest that ARGs are prevalent in the gut microbiota of these snail species and that *B. glabrata* may harbor a higher diversity and abundance of ARGs compared to *B. straminea*.

### 3.5. Potential Associations between Snail Gut Microbiota Features and Infection Rate of S. mansoni

**Figure 5:**
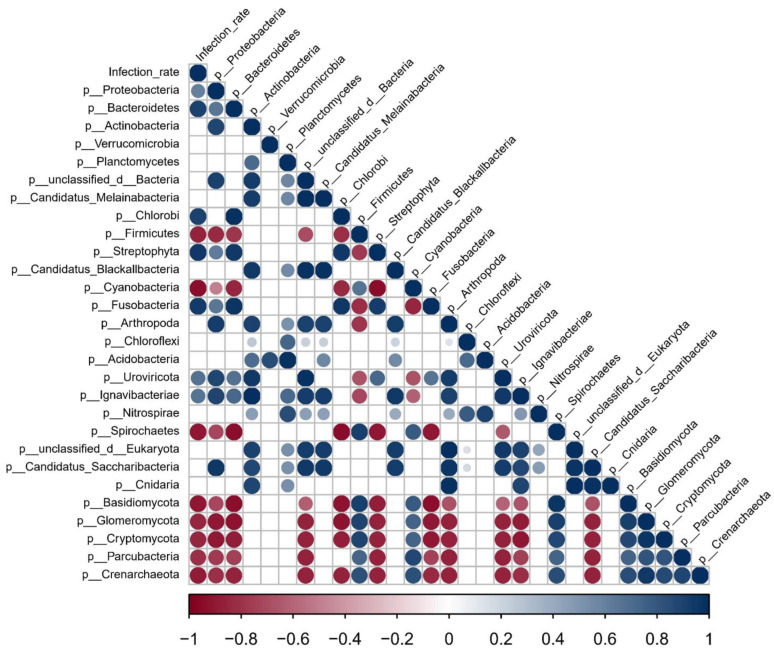
Spearman correlation shows the associations between snail gut microbiota and infection rate. The circle size indicates the magnitude of the correlation. The circles in color indicate *p* < 0.05. Specifically, the red circles represent a correlation coefficient greater than 0, while the blue circles represent a correlation coefficient less than 0. The affix “P_” indicates the phylum level.

As intermediate hosts of *S. mansoni*, *B. straminea* and *B. glabrata* play crucial roles in the transmission of *S. mansoni* [4,5,51]. However, the infection rate of *S. mansoni* in *B. glabrata* snails is significantly higher than in *B. straminea* (Figure S4). Building upon our previous findings that highlighted significant differences in gut microbiota between *B. straminea* and *B. glabrata*, we conducted a further analysis to explore the potential association between snail gut microbiota features and the infection rate of *S. mansoni*. Based on a Spearman correlation analysis, we identified a range of gut microbiota features in snails that exhibited significant correlations with the infection rate (Figure 5). For example, we observed a significant positive correlation between the abundance of the gut bacteria (Proteobacteria, Bacteroidetes and Chlorobi) and the infection rate of *S. mansoni* in snails. Conversely, we found significant negative correlations between the infection rate and the presence of Firmicutes, Cyanobacteria and Spirochaetes in the snail gut microbiota. These findings suggest that the composition and abundance of snail gut bacteria may have important implications for the infection rate of *S. mansoni*.

## 4. Discussion

Our current understanding of the gut microbiota of intermediate hosts of *S. mansoni* is limited, and we have yet to fully understand the relationship between the gut microbiota and host biology in these hosts. In this study, we conducted a metagenomic analysis to examine the difference in the gut microbiota between *B. straminea* and *B. glabrata*. Our results revealed distinct microbial compositions and functional profiles in these two host species, indicating that the host species plays a significant role in shaping the gut bacterial communities in intermediate snail hosts. Furthermore, we conducted a comprehensive investigation of ARGs in *B. straminea* and *B. glabrata*, highlighting the role of the gastropod gut microbiota as a reservoir of resistance genes and types. This study expands our knowledge of the gut microbiota in intermediate hosts and its association with antibiotic resistance in snail populations.

In our study, we first revealed the difference in the composition of gut microbes of *B. straminea* and *B. glabrata* and analyzed the relationship between gut microbes using metagenomic sequencing. Previous studies using 16S rRNA sequencing identified Proteobacteria and Bacteroidetes as dominant gut bacteria in digestive tracts of intermediate hosts such as *Pomacea canaliculata* and *Achatina fulica* and *B. glabrata* [32,52,53]. However, some snails were found to harbor a range of dominant gut microbes, including Tenericutes, Firmicutes and Proteobacteria [33]. Our findings from metagenomic analysis confirmed that Proteobacteria and Bacteroidetes are indeed the dominant gut microbes in *B. glabrata*, which is consistent with the results of previous studies [32,52,53]. Additionally, our study revealed the composition of gut microbes in intermediate hosts at the class, order, family and genus levels, respectively. At the species level, we identified specific gut microbes such as *Cloacibacterium rupense*, *Dechloromonas* sp., *Acidovorax temperans*, unclassified *Aeromonas* and *Aeromonas jandaei* that were present in *B. glabrata* but different from those in *B. straminea*. These findings provide valuable insights into the gut microbial composition of *B. straminea* and *B. glabrata*, highlighting the similarities and differences between the two species at various taxonomic levels.

We also investigated the core gut microbes of intermediate hosts using metagenomic sequencing. Core gut microbes have been identified in mammals and are known to play crucial roles in host health [18,20,48,54]. Therefore, it is important to identify the core gut microbes and understand their interactions in intermediate hosts. A previous study has defined the core microbiota of *B. glabrata* at the family level using 16S rRNA sequencing [53]. In our study, utilizing metagenomic analysis and network analysis, we revealed that the core phyla of the gut microbiota in intermediate hosts are Bacteroidetes and Proteobacteria. Furthermore, we found that core microbial genera such as *Acidovorax, Bosea*, *Aeromonas*, *Flavobacterium* and *Chryseobacterium* were associated with various microbes belonging to different clusters in intermediate hosts. Importantly, these core gut microbes in *B. straminea* and *B. glabrata* are different from those found in other animals such as fish and mosquitoes [27,55,56,57]. These findings provide valuable insights into the core gut microbes of intermediate hosts, shedding light on their associations with other microbial taxa.

The gut microbiota composition and diversity of freshwater snails can be greatly influenced by the host species. This phenomenon has been observed not only in gastropods [58] but also in vertebrates [22,24,59]. Confirming previous studies, our research using metagenomic analysis also demonstrated that variations in the gut microbiota composition and diversity of snails are indeed associated with different host species. These findings highlight the role of host species in shaping the gut microbiota of freshwater snails and support the notion that host–microbe interactions play a significant role in determining microbial community structures and diversity in intermediate hosts. Our research using metagenomic analysis also revealed that the host species has a significant influence on the gut bacterial functions of freshwater snails. Gut microbial metabolism functions play a crucial role in multiple aspects of host biology [18,21,34]. However, in gastropods, the associations between gut microbiota functions and shaping factors have not been well understood. Previous studies on the relationship between intestinal microflora and gastropod functions have mainly relied on 16S rRNA gene sequencing rather than metagenomics [33,60,61]. In our study, we compared the differences in gut microbial functions between different host species. We observed that the intermediate host *B. glabrata* exhibited significantly higher richness in functions related to metabolic pathways, biosynthesis of secondary metabolites, ABC transporters and two-component systems compared to other hosts. These findings provide important insights into the functional differences of gut microbiota in different host species, specifically highlighting the enriched metabolic and biosynthetic functions in *B. glabrata*. By utilizing metagenomic analysis, we have advanced our understanding of the complex relationships between gut microbiota and host functions in intermediate hosts. A previous study investigated the defense response and immune priming in *B*. *glabrata* and *B*. *straminea* and showed that variations in humoral defense, such as FREPs or phenoloxidase, were found between species [62]. In addition, studies on the comparisons with other mollusc genomes showed that host hormones and enzymes, such as heat shock protein and sesquiterpenoid, are extensively different between *B. straminea and B. glabrata* [63]. Therefore, host enzymes and hormones may be potential factors shaping the gut microbiota of *B*. *glabrata* and *B*. *straminea*.

Our study provided an overview of the distribution of antibiotic resistance genes and types in *B. straminea* and *B. glabrata*, highlighting the snail gut microbiome as a reservoir of resistance genes and a potential bioindicator of local antibiotic pressure. It is well established that the intestinal bacteria of various organisms harbor a significant reservoir of ARGs, which has become a global public health concern [64]. ARGs have been found to be widely distributed in the intestinal tracts of humans, mice, pigs, fish, bees and other organisms [65,66,67,68,69]. While previous studies have investigated ARGs in the intestinal flora of *B. glabrata*, they did not specifically compare the distribution of ARGs among different host species [17]. Our study, however, found that ARGs associated with various classes of antibiotics, including bacitracin, chloramphenicol, tetracycline, sulfonamide, penicillin, cephalosporin_ii and cephalosporin_i, fluoroquinolone, aminoglycoside, beta-lactam, multidrug and trimethoprim, exhibited distinctive richness in the digestive tracts of different gastropod host species. These findings enhance our understanding of the distribution of ARGs in gastropods and highlight the unique patterns of resistance genes in the gut microbiota of different host species. By comparing the ARG composition between hosts, we contribute to the knowledge of antibiotic resistance dynamics, emphasizing the importance of monitoring ARGs in the gastropod gut microbiome for assessing local antibiotic pressure.

*B. glabrata* and *B. straminea* are important vectors for the transmission of *S. mansoni* and harbor a diverse gut microbiome, as observed in the present study. It has been established that gut microbes in mosquitoes play a crucial role in preventing the transmission of pathogens [70,71,72]. In a previous study, the connection between gut bacterial dysbiosis and the snail immune response after *S. mansoni* infection was investigated [53]. However, the potential interactions among the intermediate host, its gut microbiome and the schistosome parasite remain unclear. A small number of bacterial species dominate the gut community of the host, offering advantages as models for studying gut-microbiota–host associations [73,74]. In our study, we found that *B. straminea,* which showed a lower abundance of ARGs and antibiotic resistance types in the guts, hosted a smaller scale of gut microbiota than *B. glabrata*. This suggests that *B. straminea* may be a more suitable nonmodel organism for investigating the role of gut microbiota in host–parasite interactions and host biology rather than *B. glabrata*. However, further studies are needed to fully understand these interactions. Overall, our findings shed light on the potential implications of the gut microbiota in the transmission of *S. mansoni* and provide insights into the different roles of gut microbiota in different snail species.

Moreover, our analysis indicated that there are significant associations between snail gut bacteria and the infection rate of *S. mansoni*. These findings shed light on the potential roles of gut microbiota in influencing the susceptibility of *B. straminea* and *B. glabrata* to *S. mansoni* infection. Further research in this area may provide insights into novel strategies for controlling schistosomiasis transmission by targeting the gut microbiota of snail intermediate hosts.

## 5. Conclusions

In summary, our study demonstrated that *B. straminea* and *B. glabrata* harbor different gut microbiota compositions. Host species played a significant role in shaping the community structures and functions of the gut microbiota in these intermediate hosts. We also uncovered a distinct distribution of ARGs in the gut microbiota of *B. straminea* and *B. glabrata*. Furthermore, we found correlations between snail gut microbiota and the infection rate of *S. mansoni*, suggesting a potential relationship between gut microbiota and schistosome infection. In short, these findings provide valuable insights into the interactions between gut microbiota and gastropods and may have critical consequences in terms of snail control strategies for fighting schistosomiasis in the future. Further research in this area will deepen our understanding of the complex dynamics between host, gut microbiota and parasite interactions, contributing to the development of innovative approaches for disease prevention and control.

## Data Availability

Please contact the authors for additional data requests.

## Supplementary Materials

The following supporting information can be downloaded at: https://www.mdpi.com/article/10.3390/microorganisms11102419/s1, Figure S1: The relative abundance of gut bacteria of *B. straminea* and *B. glabrata*: (A) at the class level; (B) at the order level; (C) at the family level; and (D) at the species level. The top ten most common gut microbes are shown. Figure S2: Network analysis shows the interactions between gut microbes using metagenomics analysis. (A) Phylum. (B) Genus. The edges with green represent a significant Pearson correlation. The nodes with different colors represent the microbes belonging to different clusters. *p* < 0.05 was considered statistically significant. The correlation threshold is greater than or equal to 0.5. Figure S3: The distribution of ARGs and antibiotic resistance types of the gut microbiota of *B. straminea* (n = 4) and *B. glabrata* (n = 5) are shown. (A) Heatmap displaying counts of genes belonging to antibiotic resistance genes. PCC: penicillin, cephalosporin_ii and cephalosporin_i. (B) Heatmap displaying counts of genes belonging to antibiotic resistance types. Color gradient represents the shift in antibiotic resistance genes and types. BG: *B. glabrata*. BS: *B. straminea*. Figure S4: Difference in the infection rate of *S. mansoni*-infected *B. straminea* and *B. glabrata* snails.

## Abbreviations

RH: Relative Humidity; PBS: Phosphate-Buffered Saline; ORF: Open Reading Frame; PCoA: Principal Coordinates Analysis; LEfSe: the Linear Discriminant Analysis (LDA) Effect Size; STAMP: Statistical Analysis of Metagenomic Profiles; ANOSIM: Analysis of Similarities; ARGs: Antibiotic Resistance Genes; PCC: Penicillin, Cephalosporin_ii, and Cephalosporin_i.

## References

[1] Barnett R. (2018). Schistosomiasis. Lancet.

[2] Colley D.G., Bustinduy A.L., Secor W.E., King C.H. (2014). Human schistosomiasis. Lancet.

[3] Arican-Goktas H.D., Ittiprasert W., Bridger J.M., Knight M. (2014). Differential spatial repositioning of activated genes in *Biomphalaria glabrata* snails infected with *Schistosoma mansoni*. PLoS Negl. Trop. Dis..

[4] Lin D., Xiang S., Sanogo B., Liang Y., Sun X., Wu Z. (2021). Molecular Characterization of Rotifers and Their Potential Use in the Biological Control of Biomphalaria. Front. Cell Infect. Microbiol..

[5] Lin D., Zeng X., Sanogo B., He P., Xiang S., Du S., Zhang Y., Wang L., Wan S., Zeng X. (2020). The potential risk of *Schistosoma mansoni* transmission by the invasive freshwater snail *Biomphalaria straminea* in South China. PLoS Negl. Trop. Dis..

[6] Hu Y., Chen J., Xu Y., Zhou H., Huang P., Ma Y., Gao M., Cheng S., Zhou H., Lv Z. (2020). Alterations of Gut Microbiome and Metabolite Profiling in Mice Infected by *Schistosoma japonicum*. Front. Immunol..

[7] Huang P., Zhou M., Cheng S., Hu Y., Gao M., Ma Y., Limpanont Y., Zhou H., Dekumyoy P., Cheng Y. (2020). Myricetin Possesses Anthelmintic Activity and Attenuates Hepatic Fibrosis via Modulating TGFbeta1 and Akt Signaling and Shifting Th1/Th2 Balance in *Schistosoma japonicum*-Infected Mice. Front. Immunol..

[8] Lin D., Song Q., Zhang Y., Liu J., Chen F., Du S., Xiang S., Wang L., Wu X., Sun X. (2021). *Bacillus subtilis* Attenuates Hepatic and Intestinal Injuries and Modulates Gut Microbiota and Gene Expression Profiles in Mice Infected with *Schistosoma japonicum*. Front. Cell Dev. Biol..

[9] Lin D., Song Q., Liu J., Chen F., Zhang Y., Wu Z., Sun X., Wu X. (2022). Potential Gut Microbiota Features for Non-Invasive Detection of Schistosomiasis. Front. Immunol..

[10] Madureira A.C. (2022). Programmed Cell Death-Ligand-1 expression in Bladder Schistosomal Squamous Cell Carcinoma—There’s room for Immune Checkpoint Blockage?. Front. Immunol..

[11] Trippler L., Ali S.M., Ame S.M., Hattendorf J., Suleiman K.R., Ali M.N., Juma S., Kabole F., Knopp S. (2022). Fine-scale-mapping of *Schistosoma haematobium* infections at the school and community levels and intermediate host snail abundance in the north of Pemba Island: Baseline cross-sectional survey findings before the onset of a 3-year intervention study. Parasit. Vectors.

[12] Cesari I.M., Ballén D.E., Mendoza L., Ferrer A., Pointier J.-P., Kombila M., Richard-Lenoble D., Théron A. (2014). Comparative evaluation of *Schistosoma mansoni*, *Schistosoma intercalatum*, and *Schistosoma haematobium* alkaline phosphatase antigenicity by the alkaline phosphatase immunoassay (APIA). Parasitol. Res..

[13] Chienwichai P., Tipthara P., Tarning J., Limpanont Y., Chusongsang P., Chusongsang Y., Adisakwattana P., Reamtong O. (2021). Metabolomics reveal alterations in arachidonic acid metabolism in *Schistosoma mekongi* after exposure to praziquantel. PLoS Negl. Trop. Dis..

[14] Rahman M.O., Sassa M., Parvin N., Islam M.R., Yajima A., Ota E. (2021). Diagnostic test accuracy for detecting *Schistosoma japonicum* and *S. mekongi* in humans: A systematic review and meta-analysis. PLoS Negl. Trop. Dis..

[15] Chienwichai P., Nogrado K., Tipthara P., Tarning J., Limpanont Y., Chusongsang P., Chusongsang Y., Tanasarnprasert K., Adisakwattana P., Reamtong O. (2022). Untargeted serum metabolomic profiling for early detection of *Schistosoma mekongi* infection in mouse model. Front. Cell Infect. Microbiol..

[16] Brodin P. (2022). Immune-microbe interactions early in life: A determinant of health and disease long term. Science.

[17] Du S., Sun X., Zhang J., Lin D., Chen R., Cui Y., Xiang S., Wu Z., Ding T. (2022). Metagenome-Assembled Genomes Reveal Mechanisms of Carbohydrate and Nitrogen Metabolism of Schistosomiasis-Transmitting Vector Biomphalaria Glabrata. Microbiol. Spectr..

[18] Chevalier C., Stojanović O., Colin D.J., Suarez-Zamorano N., Tarallo V., Veyrat-Durebex C., Rigo D., Fabbiano S., Stevanović A., Hagemann S. (2015). Gut Microbiota Orchestrates Energy Homeostasis during Cold. Cell.

[19] Videvall E., Song S.J., Bensch H.M., Strandh M., Engelbrecht A., Serfontein N., Hellgren O., Olivier A., Cloete S., Knight R. (2020). Early-life gut dysbiosis linked to juvenile mortality in ostriches. Microbiome.

[20] Zhang X.Y., Sukhchuluun G., Bo T.B., Chi Q.S., Yang J.J., Chen B., Zhang L., Wang D.-H. (2018). Huddling remodels gut microbiota to reduce energy requirements in a small mammal species during cold exposure. Microbiome.

[21] Zhang Z., Xu D., Wang L., Hao J., Wang J., Zhou X., Wang W., Qiu Q., Huang X., Zhou J. (2016). Convergent Evolution of Rumen Microbiomes in High-Altitude Mammals. Curr. Biol..

[22] Minich J.J., Härer A., Vechinski J., Frable B.W., Skelton Z.R., Kunselman E., Shane M.A., Perry D.S., Gonzalez A., McDonald D. (2022). Host biology, ecology and the environment influence microbial biomass and diversity in 101 marine fish species. Nat. Commun..

[23] Baniel A., Amato K.R., Beehner J.C., Bergman T.J., Mercer A., Perlman R.F., Petrullo L., Reitsema L., Sams S., Lu A. (2021). Seasonal shifts in the gut microbiome indicate plastic responses to diet in wild geladas. Microbiome.

[24] Kim P.S., Shin N.R., Lee J.B., Kim M.S., Whon T.W., Hyun D.W., Yun J.-H., Jung M.-J., Kim Y.J., Bae J.W. (2021). Host habitat is the major determinant of the gut microbiome of fish. Microbiome.

[25] Li F., Li C., Chen Y., Liu J., Zhang C., Irving B., Fitzsimmons C., Plastow G., Guan L.L. (2019). Host genetics influence the rumen microbiota and heritable rumen microbial features associate with feed efficiency in cattle. Microbiome.

[26] Luo T., Li Y., Zhang W., Liu J., Shi H. (2022). Rumen and fecal microbiota profiles associated with immunity of young and adult goats. Front. Immunol..

[27] Ranasinghe K., Gunathilaka N., Amarasinghe D., Rodrigo W., Udayanga L. (2021). Diversity of midgut bacteria in larvae and females of *Aedes aegypti* and *Aedes albopictus* from Gampaha District, Sri Lanka. Parasit. Vectors.

[28] Wan S., Sun X., Wu F., Yu Z., Wang L., Lin D., Li Z., Wu Z., Sun X. (2018). Chi3l3: A potential key orchestrator of eosinophil recruitment in meningitis induced by *Angiostrongylus cantonensis*. J. Neuroinflammation..

[29] Ji L., Yiyue X., Xujin H., Minghui Z., Mengying Z., Yue H., Yanqi W., Langui S., Xin Z., Datao L. (2017). Study on the tolerance and adaptation of rats to *Angiostrongylus cantonensis* infection. Parasitol. Res..

[30] Song L.G., Zheng X.Y., Lin D.T., Wang G.X., Wu Z.D. (2017). Parasitology should not be abandoned: Data from outpatient parasitological testing in Guangdong, China. Infect. Dis. Poverty..

[31] Pinheiro G.L., Correa R.F., Cunha R.S., Cardoso A.M., Chaia C., Clementino M.M., Garcia E.S., de Souza W., Frasés S. (2015). Isolation of aerobic cultivable cellulolytic bacteria from different regions of the gastrointestinal tract of giant land snail *Achatina fulica*. Front. Microbiol..

[32] Li L.-H., Lv S., Lu Y., Bi D.-Q., Guo Y.-H., Wu J.-T., Yue Z.-Y., Mao G.-Y., Guo Z.-X., Zhang Y. (2019). Spatial structure of the microbiome in the gut of *Pomacea canaliculata*. BMC Microbiol..

[33] Chen L., Li S., Xiao Q., Lin Y., Li X., Qu Y., Wu G., Li H. (2021). Composition and diversity of gut microbiota in *Pomacea canaliculata* in sexes and between developmental stages. BMC Microbiol..

[34] Jiang Q., Lin L., Xie F., Jin W., Zhu W., Wang M., Qiu Q., Li Z., Liu J., Mao S. (2022). Metagenomic insights into the microbe-mediated B and K(2) vitamin biosynthesis in the gastrointestinal microbiome of ruminants. Microbiome.

[35] Salazar C., Giménez M., Riera N., Parada A., Puig J., Galiana A., Grill F., Vieytes M., Mason C.E., Antelo V. (2022). Human microbiota drives hospital-associated antimicrobial resistance dissemination in the urban environment and mirrors patient case rates. Microbiome.

[36] Zhang X.-L., Deng Y.-P., Yang T., Li L.-Y., Cheng T.-Y., Liu G.-H., Duan D.-Y. (2022). Metagenomics of the midgut microbiome of *Rhipicephalus microplus* from China. Parasit. Vectors.

[37] Isaac S., Flor-Duro A., Carruana G., Puchades-Carrasco L., Quirant A., Lopez-Nogueroles M., Pineda-Lucena A., Garcia-Garcera M., Ubeda C. (2022). Microbiome-mediated fructose depletion restricts murine gut colonization by vancomycin-resistant *Enterococcus*. Nat. Commun..

[38] Qu G., Wang W., Lu X., Dai J., Li X., Liang Y. (2016). Evaluating the risk of *Schistosoma mansoni* transmission in mainland China. Parasitol. Res..

[39] Li H., Durbin R. (2009). Fast and accurate short read alignment with Burrows-Wheeler transform. Bioinformatics.

[40] Noguchi H., Park J., Takagi T. (2006). MetaGene: Prokaryotic gene finding from environmental genome shotgun sequences. Nucleic Acids Res..

[41] Buchfink B., Xie C., Huson D.H. (2015). Fast and sensitive protein alignment using DIAMOND. Nat. Methods.

[42] Avershina E., Frisli T., Rudi K. (2013). De novo semi-alignment of 16S rRNA gene sequences for deep phylogenetic characterization of next generation sequencing data. Microbes Environ..

[43] Segata N., Izard J., Waldron L., Gevers D., Miropolsky L., Garrett W.S., Huttenhower C. (2011). Metagenomic biomarker discovery and explanation. Genome Biol..

[44] Fernandez M.A., Thiengo S.C. (2002). Susceptibility of Biomphalaria straminea (Dunker, 1848) from Serra da Mesa Dam, Goias, Brazil to infection with three strains of *Schistosoma mansoni* Sambon, 1907. Mem. Inst. Oswaldo. Cruz..

[45] Wang Z., Klipfell E., Bennett B.J., Koeth R., Levison B.S., DuGar B., Feldstein A.E., Britt E.B., Fu X., Chung Y.-M. (2011). Gut flora metabolism of phosphatidylcholine promotes cardiovascular disease. Nature.

[46] Tremaroli V., Backhed F. (2012). Functional interactions between the gut microbiota and host metabolism. Nature.

[47] Wang Y., Zhou J., Ye J., Sun Z., He Y., Zhao Y., Ren S., Zhang G., Liu M., Zheng P. (2023). Multi-omics reveal microbial determinants impacting the treatment outcome of antidepressants in major depressive disorder. Microbiome.

[48] Wang X., Tsai T., Deng F., Wei X., Chai J., Knapp J., Apple J., Maxwell C.V., Lee J.A., Li Y. (2019). Longitudinal investigation of the swine gut microbiome from birth to market reveals stage and growth performance associated bacteria. Microbiome.

[49] Bengtsson-Palme J., Larsson D.G. (2015). Antibiotic resistance genes in the environment: Prioritizing risks. Nat. Rev. Microbiol..

[50] Young C.C.W., Karmacharya D., Bista M., Sharma A.N., Goldstein T., Mazet J.A.K., Johnson C.K. (2022). Antibiotic resistance genes of public health importance in livestock and humans in an informal urban community in Nepal. Sci. Rep..

[51] Adema C.M., Hillier L.W., Jones C.S., Loker E.S., Knight M., Minx P., Oliveira G., Raghavan N., Shedlock A., do Amaral L.R. (2017). Whole genome analysis of a schistosomiasis-transmitting freshwater snail. Nat. Commun..

[52] Osório J.B., Pereira L.d.M., Giongo A., Marconatto L., Potriquet J., Candido R.R.F., Mulvenna J., Jones M., Graeff-Teixeira C., Morassutti A.L. (2020). Mollusk microbiota shift during *Angiostrongylus cantonensis* infection in the freshwater snail *Biomphalaria glabrata* and the terrestrial slug *Phillocaulis soleiformis*. Parasitol. Res..

[53] Portet A., Toulza E., Lokmer A., Huot C., Duval D., Galinier R., Gourbal B. (2021). Experimental Infection of the *Biomphalaria glabrata* Vector Snail by *Schistosoma mansoni* Parasites Drives Snail Microbiota Dysbiosis. Microorganisms.

[54] Worthmann A., John C., Rühlemann M.C., Baguhl M., Heinsen F.-A., Schaltenberg N., Heine M., Schlein C., Evangelakos I., Mineo C. (2017). Cold-induced conversion of cholesterol to bile acids in mice shapes the gut microbiome and promotes adaptive thermogenesis. Nat. Med..

[55] Lin D., Zheng X., Sanogo B., Ding T., Sun X., Wu Z. (2021). Bacterial composition of midgut and entire body of laboratory colonies of *Aedes aegypti* and *Aedes albopictus* from Southern China. Parasit. Vectors.

[56] Kokou F., Sasson G., Friedman J., Eyal S., Ovadia O., Harpaz S., Cnaani A., Mizrahi I. (2019). Core gut microbial communities are maintained by beneficial interactions and strain variability in fish. Nat. Microbiol..

[57] Stephens W.Z., Burns A.R., Stagaman K., Wong S., Rawls J.F., Guillemin K., Bohannan B.J.M. (2016). The composition of the zebrafish intestinal microbial community varies across development. ISME J..

[58] Cicala F., Cisterna-Celiz J.A., Moore J.D., Rocha-Olivares A. (2018). Structure, dynamics and predicted functional role of the gut microbiota of the blue (*Haliotis fulgens*) and yellow (*H. corrugata*) abalone from Baja California Sur, Mexico. PeerJ.

[59] Wang Z., Zhang C., Li G., Yi X. (2022). The influence of species identity and geographic locations on gut microbiota of small rodents. Front. Microbiol..

[60] Lyu T., Zhu J., Yang X., Yang W., Zheng Z. (2022). Responses of Gut Microbial Community Composition and Function of the Freshwater Gastropod Bellamya aeruginosa to Cyanobacterial Bloom. Front. Microbiol..

[61] Hao Y., Guan W., Wu H., Li L., Abe E.M., Xue J., Qin Z., Wang Q., Lv S., Xu J. (2020). Intestinal microbiome profiles in *Oncomelania hupensis* in mainland China. Acta Trop..

[62] de Melo E.S., Brayner F.A., Junior N., Franca I., Alves L.C. (2020). Investigation of defense response and immune priming in *Biomphalaria glabrata* and *Biomphalaria straminea*, two species with different susceptibility to *Schistosoma mansoni*. Parasitol. Res..

[63] Nong W., Yu Y., Aase-Remedios M.E., Xie Y., So W.L., Li Y., Wong C.F., Baril T., Law S.T.S., Lai S.Y. (2022). Genome of the ramshorn snail *Biomphalaria straminea*—An obligate intermediate host of schistosomiasis. Gigascience.

[64] Cui E., Zhou Z., Gao F., Chen H., Li J. (2023). Roles of substrates in removing antibiotics and antibiotic resistance genes in constructed wetlands: A review. Sci. Total Environ..

[65] Sun H., Mu X., Zhang K., Lang H., Su Q., Li X., Zhou X., Zhang X., Zheng H. (2022). Geographical resistome profiling in the honeybee microbiome reveals resistance gene transfer conferred by mobilizable plasmids. Microbiome.

[66] Hu Y., Yang X., Qin J., Lu N., Cheng G., Wu N., Pan Y., Li J., Zhu L., Wang X. (2013). Metagenome-wide analysis of antibiotic resistance genes in a large cohort of human gut microbiota. Nat. Commun..

[67] Wang Y., Hu Y., Liu F., Cao J., Lv N., Zhu B., Zhang G., Gao G.F. (2020). Integrated metagenomic and metatranscriptomic profiling reveals differentially expressed resistomes in human, chicken, and pig gut microbiomes. Environ. Int..

[68] Tan R., Jin M., Shao Y., Yin J., Li H., Chen T., Shi D., Zhou S., Li J., Yang D. (2022). High-sugar, high-fat, and high-protein diets promote antibiotic resistance gene spreading in the mouse intestinal microbiota. Gut Microbes..

[69] Zhang M., Hou L., Zhu Y., Zhang C., Li W., Lai X., Yang J., Li S., Shu H. (2022). Composition and distribution of bacterial communities and antibiotic resistance genes in fish of four mariculture systems. Environ. Pollut..

[70] Dutra H.L., Rocha M.N., Dias F.B., Mansur S.B., Caragata E.P., Moreira L.A. (2016). Wolbachia Blocks Currently Circulating Zika Virus Isolates in Brazilian *Aedes aegypti* Mosquitoes. Cell Host Microbe..

[71] Wang S., Dos-Santos A.L.A., Huang W., Liu K.C., Oshaghi M.A., Wei G., Agre P., Jacobs-Lorena M. (2017). Driving mosquito refractoriness to *Plasmodium falciparum* with engineered symbiotic bacteria. Science.

[72] Dong Y., Manfredini F., Dimopoulos G. (2009). Implication of the mosquito midgut microbiota in the defense against malaria parasites. PLoS Pathog..

[73] Liberti J., Engel P. (2020). The gut microbiota—Brain axis of insects. Curr. Opin. Insect Sci..

[74] Nagpal J., Cryan J.F. (2021). Microbiota-brain interactions: Moving toward mechanisms in model organisms. Neuron.

