# Supplementary material for: Metagenomic Analysis Reveals Variations in Gut Microbiomes of the Schistosoma mansoni-Transmitting Snails Biomphalaria straminea and Biomphalaria glabrata
Source: Microorganisms. 2023 Sep 28;11(10):2419. doi: 10.3390/microorganisms11102419 (PMC10609589; doi:10.3390/microorganisms11102419)
Supplement: Supplementary file 1 [file microorganisms-11-02419-s001.zip › microorganisms-2569406-supplementary.pdf]

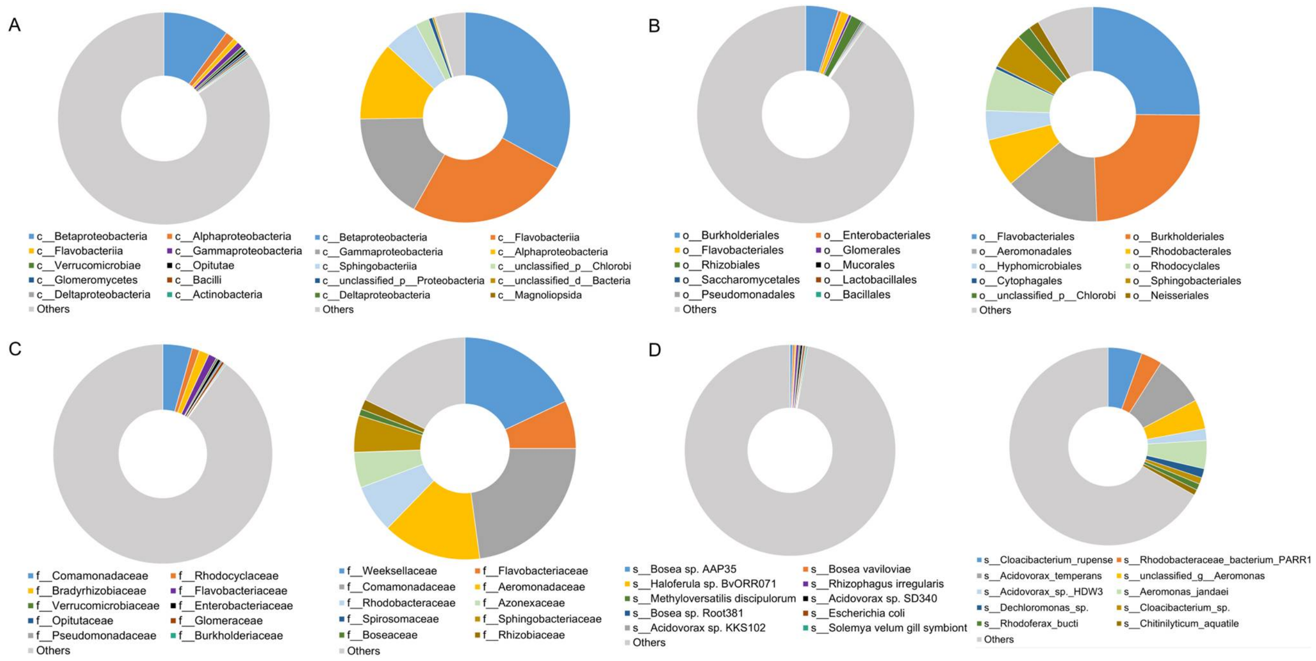

Figure S1. The relative abundance of gut bacteria of *B. straminea* and *B. glabrata*. (**A**) At the class level. (**B**) At the order level. (**C**) At the family level. (**D**) At the species level. The top ten of the most common gut microbes are shown.

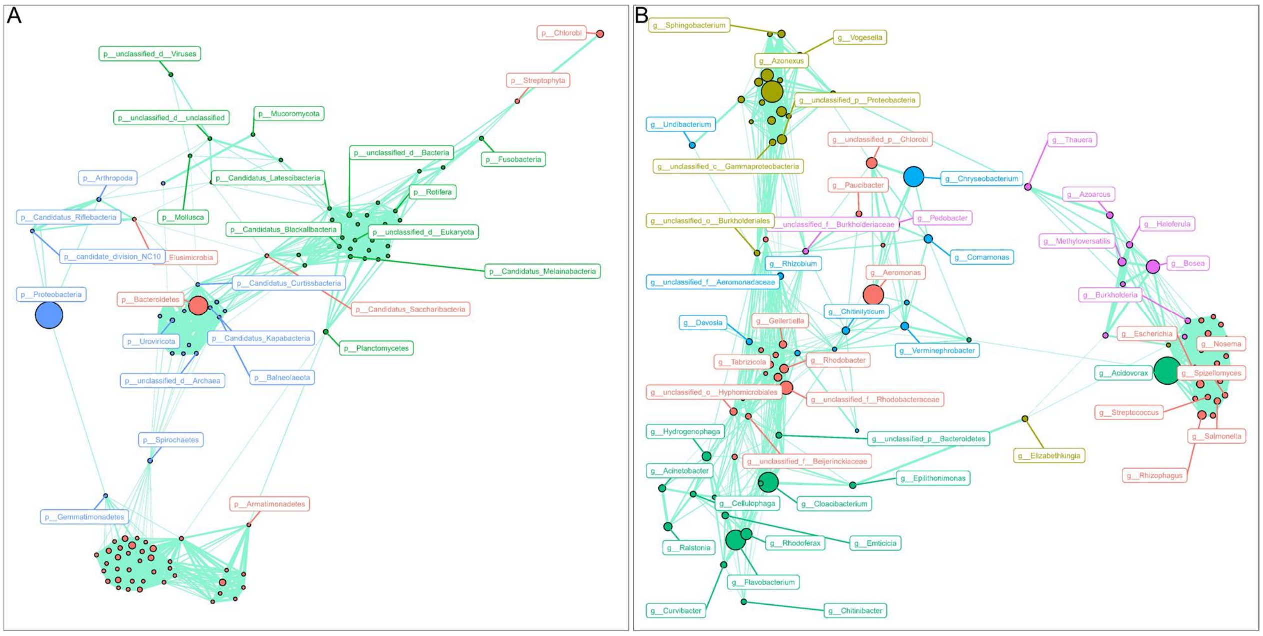

Figure S2. Network analysis shows the interactions between gut microbes using metagenomics analysis. (**A**) Phylum. (**B**) Genus. The edges with green represent a significant Pearson correlation. The nodes with different colours represent the microbes belonging to different clusters. *P* <0.05 were considered statistically significant. The correlation threshold is greater than or equal to 0.5.

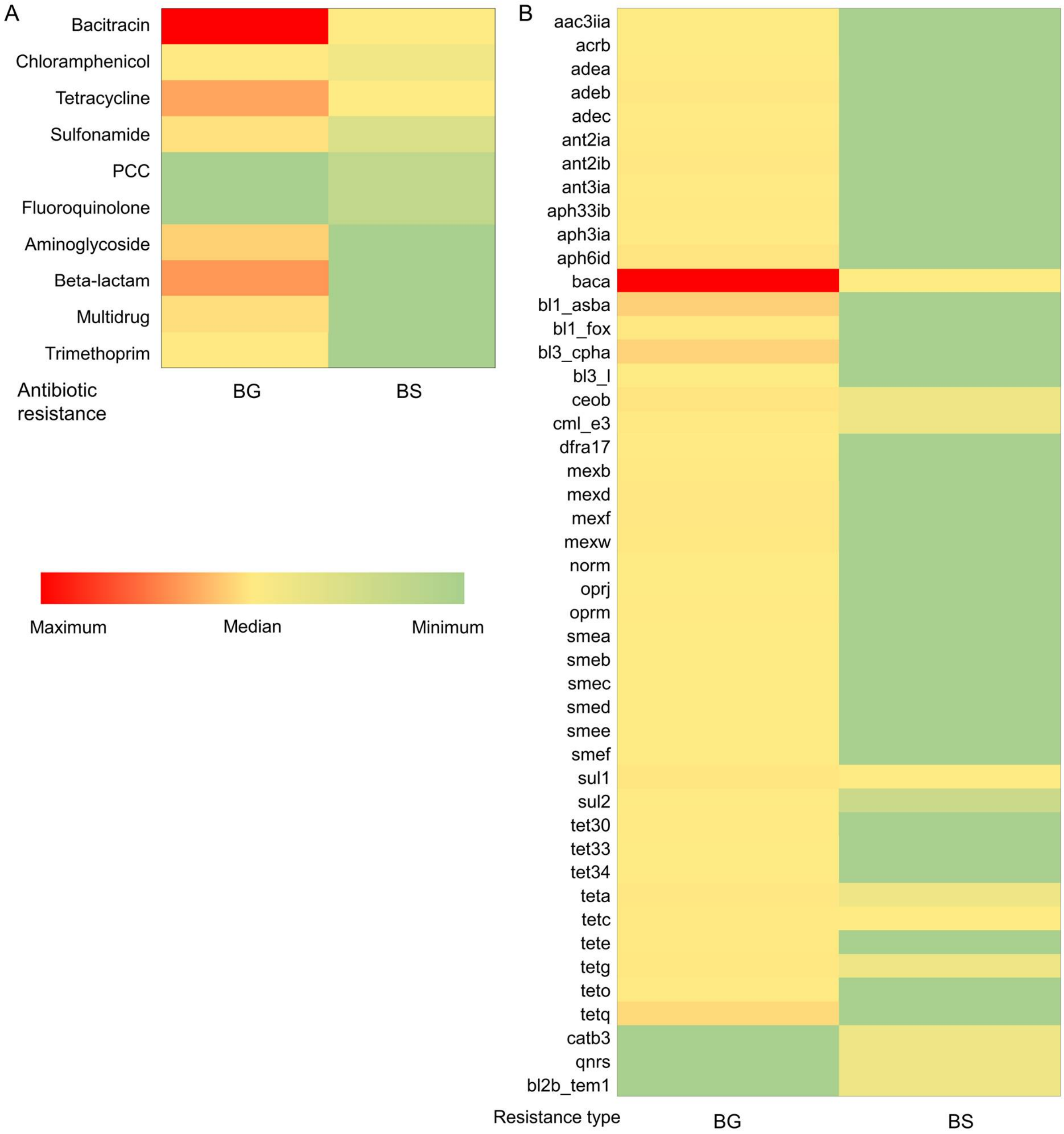

Figure S3. The distribution of ARGs and antibiotic resistance types of the gut microbiota of *B. straminea* (n =4) and *B. glabrata* (n =5) are shown. (**A**) Heatmap displaying counts of genes belonging to antibiotic resistance genes. PCC: Penicillin, cephalosporin_ii, cephalosporin_i. (**B**) Heatmap displaying counts of genes belonging to antibiotic resistance types. Color gradient represents the shift of antibiotic resistance genes and types. BG: *B. glabrata*. BS: *B. straminea*.

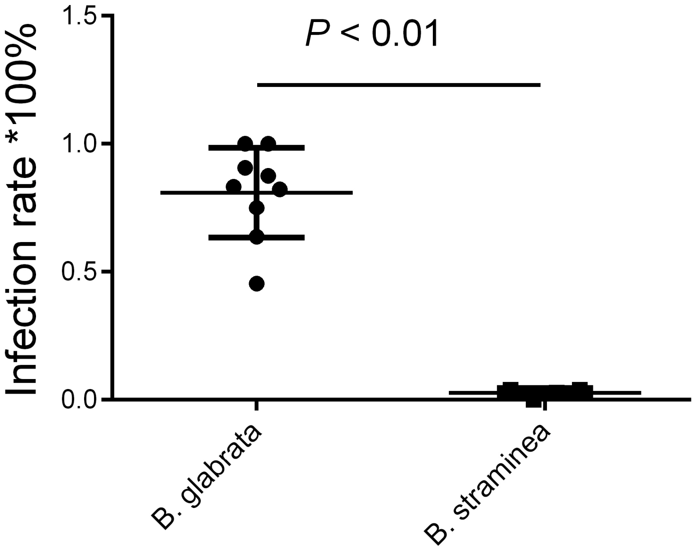

Figure S4. Difference in the infection rate of *S. mansoni*-infected *B. straminea* and *B. glabrata* snails.
